# Effects of perceived cocaine availability on subjective and objective responses to the drug

**DOI:** 10.1186/1747-597X-2-30

**Published:** 2007-10-11

**Authors:** Rinah T Yamamoto, Katherine H Karlsgodt, David Rott, Scott E Lukas, Igor Elman

**Affiliations:** 1 Behavioral Psychopharmacology Research Laboratory, McLean Hospital/Harvard Medical School, 115 Mill St., Belmont, MA, USA; 2 Addiction Research Program, Massachusetts General Hospital/Harvard Medical School, Department of Psychiatry, 15 Parkman St., Boston MA USA; 3 Department of Medicine, Hadassah-Hebrew University Medical Center, Israel; 4 Department of Psychology, University of California, Los Angeles, CA, USA

## Abstract

**Rationale:**

Several lines of evidence suggest that cocaine expectancy and craving are two related phenomena. The present study assessed this potential link by contrasting reactions to varying degrees of the drug's perceived availability.

**Method:**

Non-treatment seeking individuals with cocaine dependence were administered an intravenous bolus of cocaine (0.2 mg/kg) under 100% ('unblinded'; N = 33) and 33% ('blinded'; N = 12) probability conditions for the delivery of drug. Subjective ratings of craving, high, rush and low along with heart rate and blood pressure measurements were collected at baseline and every minute for 20 minutes following the infusions.

**Results:**

Compared to the 'blinded' subjects, their 'unblinded' counterparts had similar craving scores on a multidimensional assessment several hours before the infusion, but reported higher craving levels on a more proximal evaluation, immediately prior to the receipt of cocaine. Furthermore, the 'unblinded' subjects displayed a more rapid onset of high and rush cocaine responses along with significantly higher cocaine-induced heart rate elevations.

**Conclusion:**

These results support the hypothesis that cocaine expectancy modulates subjective and objective responses to the drug. Provided the important public health policy implications of heavy cocaine use, health policy makers and clinicians alike may favor cocaine craving assessments performed in the settings with access to the drug rather than in more neutral environments as a more meaningful marker of disease staging and assignment to the proper level of care.

## 1. Background

Cocaine dependence is a chronically relapsing disorder leading to a variety of medical complications along with devastating psychosocial consequences. It remains a major public health problem bearing enormous societal costs and is currently afflicting over 1.5 million American citizens [1]. Thus, epidemiological data presented at the recent Community Epidemiology Work Group meeting indicate that in a number of major US cities, representing 21 geographic areas, hospital admissions for primary cocaine-related problems exceeded those for heroin, methamphetamine and marihuana, altogether [2].

Craving, defined as an intense and often irrepressible urge to seek and consume the drug, resulting in relapse even after extended periods of abstinence [3], is one of the most malignant and treatment resistant features of cocaine dependence [4]. Therefore, in the search for innovative and efficient therapeutic approaches, craving has been subjected to an intensive scientific inquiry in both laboratory and clinical arenas. One consistent finding produced by this effort, is a considerable overlap between neural correlates of craving, cognition and motivation [3,5-7]. Hence, understanding how cocaine produces its effects necessitates investigation of the cognitive and motivational contexts of craving.

Cocaine craving may result from the desire for pleasurable effects of the drug [8]. A closely related concept to this model is that of dopamine as the motivation/cognition-related neurotransmitter, the phasic release of which underlies the anticipation of pleasurable outcomes and motivational behaviors targeted at avoiding the loss of pleasure [9-16].

Primate electrophysiological studies of individual dopamine neurons in the brain [9,17-21] have begun dissecting these motivational processes that could be potentially broadened to cocaine craving. Neuronal responses to reward and reward-predicating stimuli appear to be dependent on event predictability i.e., neuronal activity is increased by events with reward values which are better than predicted, is uninfluenced by events that are as good as predicted, and is reduced by events that are worse than predicted [13,22,23]. Elevated dopamine concentrations may also prime the organism to associate cues around cocaine use with the pleasure experienced when drug is taken [12,13,15,18-20,24]. When these cues are encountered again, they may elicit a surge of activity in dopaminergic neurons leading to exaggerated anticipatory craving of the future cocaine reward [25]. However, if individuals with cocaine dependence are exposed to stimuli associated with cocaine when they cannot consume it, they may experience a decrease in dopamine activity, which may potentially lead to a dysphoric state of withdrawal [16].

These observations lead Volkow and colleagues [16] to assume the existence of specific neural loci related to the processes of expectancy, predictions of reward and contingency assessment. They assessed this entity by using a two-by-two within subjects design to measure the effects of expectation on subjective effects and regional brain metabolism in cocaine abusers under four conditions: 1) expecting and receiving placebo; 2) expecting placebo and receiving a drug similar to cocaine, methylphenidate; 3) expecting methylphenidate and receiving placebo; 4) expecting and receiving methylphenidate. The authors found that expectation of the drug and subsequent receipt of the drug led to an increased physiological response. This was reflected in a 50% increase in global brain glucose utilization, with specific increases in the thalamic and cerebellar regions and an enhancement in subjective assessments ("drug liking," "high," "feel drug," and "restlessness") compared to expectation of placebo and receipt of drug [16]. Thus, after methylphenidate infusion, the presence or absence of the drug-induced subjective effects apparently involved some retrieval and comparison to the "remembered utility" [16], or the emotional memory associated with the prior use.

The present study attempted to extend Volkow et al.'s [16] findings to cocaine itself and to craving. To that end, cocaine dependent subjects' self-reports of craving when they were certain in the receipt of the drug were contrasted to those when such probability stood at only 33%. The primary hypothesis to be tested was that subjects experience stronger craving when they know that cocaine infusion is forthcoming versus a situation in which it is less likely. Other subjective ratings i.e., high, rush and low [16,26] were assessed using exploratory analysis. Finally, given the previous finding by our group that subjective expectancy ratings accounted for some variance in the hemodynamic responses to 'unblinded' cocaine [27], we also examined the effects of perceived cocaine availability on heart rate and on mean arterial pressure (MAP).

## 2. Methods

### 2.1 Participants

The study samples were comprised of non-treatment seeking cocaine-dependent participants recruited by advertisement who received cocaine under 'unblinded' (N = 33; 28 males and 5 females, 23 Caucasian and 10 African-American; mean age ± SD: 34.2 ± 6.0 years; weight: 77.0 ± 15.2 kg) and 'blinded' conditions (9 males and 3 females; 4 Caucasian and 8 African-American; mean age: 39.7 ± 7.8 years; weight: 79.9 ± 13.6 kg). The subjects were in good physical health, not taking prescription medications or other illegal drugs. All met DSM-IV criteria for cocaine dependence with no past or current major depression or other Axis I psychiatric diagnosis as assessed by the Structured Clinical Interview for DSM-IV Axis I Disorders (SCID; [28]). Individuals using prescription medications or those with dependence on other illegal drugs or alcohol were excluded from study participation. 'Unblinded' and 'blinded' subjects' cocaine use averaged 13.2 ± 7.0 and 6.5 ± 7.4 (t_43 _= 0.13, *p *= 0.9) times per month with the last cocaine use 1.7 ± 1.2 and 1.4 ± 1.1 (t_43 _= 0.76, *p *= 0.45) days prior to study participation, respectively, with smoking being the primary route of cocaine ingestion. Subjects provided written informed consent for the Massachusetts General Hospital (MGH) Institutional Review Board-approved protocol prior to initiating the study. The data from these samples were previously reported [29-31] and are included here for the purpose of contrasting the two expectancy states (i.e., 'unblinded' vs. 'blinded').

### 2.2 Clinical protocol

Subjects were admitted to the MGH General Clinical Research Center, having abstained from cocaine for at least 10 hours and completed medical workup, including urinalysis and breathalyzer tests for drug use, and structured clinical assessments including SCID [28], Addiction Severity Index (ASI; [32]) and a multidimensional craving questionnaire [33,34].

The latter assessment tool was demonstrated to be a reliable predictor of short-, but not long-term cocaine use [33,34] and it measures various aspects of craving on a Likert-type scale (items rated on a scale of 0–9), including (i) current intensity; (ii) desire to avoid using; (iii) capacity to resist using; (iv) responsiveness to drug-related conditioned stimuli; and (v) imagined likelihood of use if in a setting with access to drugs. Subjects were assessed for spontaneous craving after at least 10 hours abstinence prior to administration of the infusions. Total spontaneous craving score was derived by adding together ratings scores on items 1, 4 and 5 and subtracting items 2 and 3 [35]. The 'unblinded' and 'blinded' groups were comparable in terms of their ASI drug composite scores (0.2 ± 0.1 and 0.2 ± 0.1; t_43 _= 0.2, *p *= 0.9) and the craving ratings the total scores (17.6 ± 10.3 and 16.6 ± 10.5, respectively; t_43 _= 0.3, *p *= 0.8) on the multidimensional craving questionnaire.

For the 'unblinded' condition, the subjects were informed that they would receive 0.2 mg/kg of cocaine, whereas the 'blinded' subjects were told they could receive any of three infusions, namely cocaine 0.2 mg/kg, a low dose of hydrocortisone or a placebo and that each subsequent infusion was non-contingent on the experience with the one preceding it.

Bilateral antecubital intravenous catheters were placed (left forearm for infusions, right forearm for serial blood sampling) and the infusion(s) was/were commenced after a 60-minute rest period. Cocaine, hydrocortisone and saline were administered as intravenous boluses in a volume of 10 mL, 2 hours apart in a double-blind, randomized and counterbalanced cross-over design. Only cocaine data are included in this report; hydrocortisone and saline results are presented elsewhere [29-31]. Continuous hemodynamic monitoring was performed using OmniTrak 3100 patient monitoring system (Orlando, FL) and a board-certified cardiologist was present throughout the whole study.

### 2.3 Subjective self-reports

The individualized description of subjective responses were categorized into 4 components : craving, high, rush, and low [36]. Cocaine craving was defined proactively with each subject; clinically, as an urge to use the drug and operationally, in terms of the action the individual would be willing to engage in order to get more cocaine. The following were subjectively defined, but not necessarily associated with a behavioral response or with the planning of physical activity: high (well-being, self-confidence, and sociability), rush (perception of elevated heart rate and sweating, along with sensations of "speeding") and low (dysphoric affect distinct from high experience diminishment) [36]. Thus, of the four measures, only craving was associated with a behavioral response or with planning or implementation of physical activity. Therefore, by definition, only craving self-report could be defined as a motivational state [36]. Behavioral ratings were acquired using a Macintosh laptop placed on a table in front of the subject. The words "CRAVING", "HIGH", "RUSH" and "LOW" appeared sequentially on the screen and with each appearance the subject used the keyboard to rate them on a continuous Likert-type scale of 0 (none) to 3 (extreme). Ratings were initiated 2 minutes pre-infusion and a full set was collected once per minute until 20 minutes post-infusion.

### 2.4 Data analysis

Data were analyzed using SPSS 13.0 for Mac (SPSS, Inc., Chicago, IL). Baseline subjective scores were determined from the mean of the self-reported ratings at 1- and 2-minute time points prior to cocaine administration and were compared using Student's t-test. When variances were determined to be dissimilar, t-test corrected for separate variances was used. Repeated measures ANOVAs – 2 (group: 'unblinded' and 'blinded') × 20 (time: 20 minutes) were used to assess the subjective- and hemodynamic variables. When violations of sphericity/homogeneity assumptions were determined, a stringent Greenhouse-Geisser (GG) correction was applied. In light of previously reported cocaine's subjective effects in both groups (Elman et al., 2002; Elman et al., 2003), *a priori *emphasis was given to the group by time interaction effect comparing responses of the 'unblinded' and 'blinded' groups. Data were summarized as Mean ± standard deviation. All analyses were two-tailed with α < 0.05 set as the threshold for statistical significance.

## 3. Results

### 3.1 Covariation

Subjects' age was used as a covariate because participants in the 'unblinded' group were younger (t_43 _= 2.47, *p *< 0.02) than in the 'blinded' group. To adjust for variability in the baseline measures, subjective and hemodynamic data were also (in addition to age) covaried for the pre-infusion baseline values, as appropriate (e.g., baseline craving reports for the craving data).

### 3.2 Subjective responses

**Figure 1:**
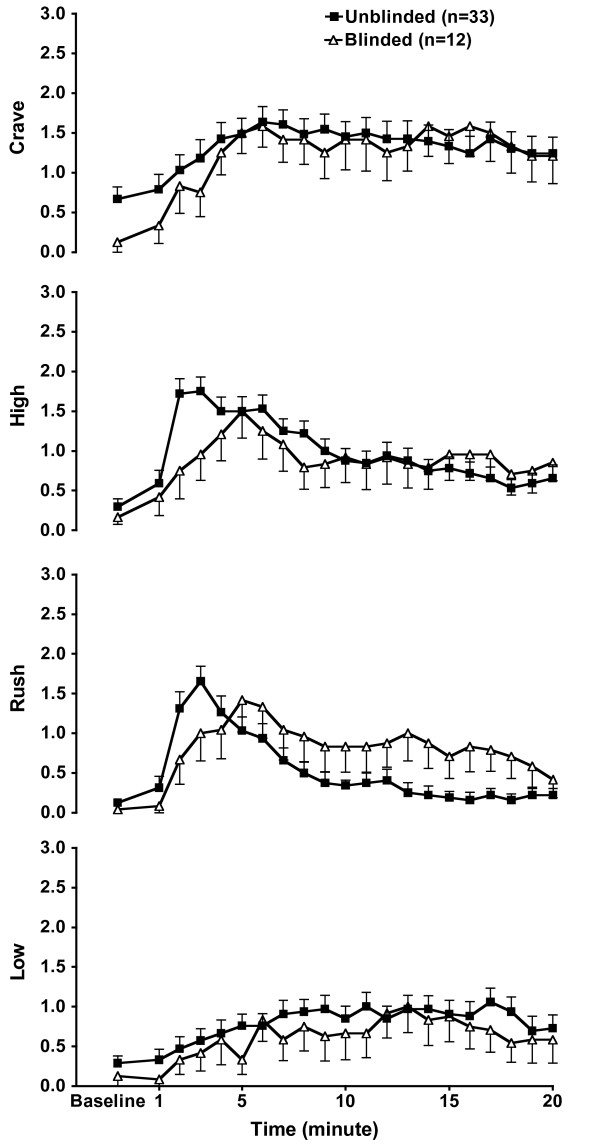
**Subjective responses**. Effects of 'unblinded' vs. 'blinded' cocaine administration on craving, high rush and low ratings. Data are presented as mean (± SEM).

Subjective data are graphically presented in Figure 1. Despite similar craving scores on the multidimensional craving questionnaire, pre-infusion craving ratings were significantly higher (t_43 _= 2.05, corrected *p *< 0.009) in the 'unblinded' subjects, (0.67 ± 0.9), as compared to their 'blinded' counterparts (0.13 ± 0.4). In contrast, there were no significant group differences in the pre-infusion high, rush, or low ratings (*p *> 0.35).

For craving, repeated-measures ANOVA, covaried for the baseline self-rating scores and age, revealed no effect of group or group by time interaction (F_1,41 _= 1.8, *p *> 0.20), indicating that following cocaine administration, both groups' relative craving levels increased in a similar fashion across the study time frame, with a main effect for time (F_19,760 _= 3.21, GG adjusted *p *< 0.02).

For high, the group by time interaction was significant (F_19,760 _= 2.09, *p *< 0.004), this effect, however, was not sustained with the GG correction (adjusted *p *= 0.06). Rush ratings differences reached statistical significance resulting in significant group by time interaction (F_19,760 _= 3.37, GG adjusted *p *< 0.004). Group differences in the low ratings did not reach statistical significance (F_19,760 _= 0.55, GG adjusted *p *= 0.73).

### 3.3 Hemodynamic responses

**Figure 2:**
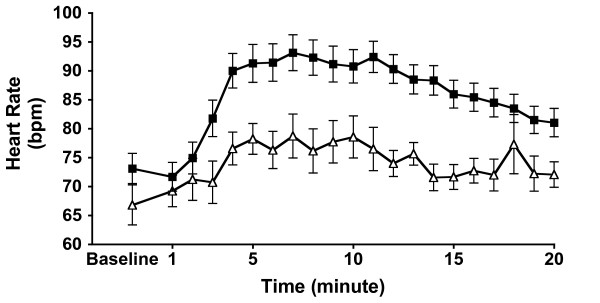
**Heart rate**. Effects of 'unblinded' vs. 'blinded' cocaine administration on mean arterial pressure and heart rate. Data are presented as mean (± SEM).

Cocaine produced significantly higher heart rate increases in the 'unblinded' subjects (Figure 2) (group effect: F_1,41 _= 7.26, *p *< 0.01; group-by-time interaction: F_19,779 _= 3.25, GG adjusted *p *< 0.006; mean change from baseline: 30% versus 20%). Changes in MAP were not apparent across time by group (F_19,779 _= 1.63, GG adjusted *p *= 0.12).

## 4. Discussion

The major finding of this study is that changing the motivational context from 100% to 33% cocaine expectancy, by matching a potential cocaine infusion to two other potential infusions, significantly modified subjective and objective cocaine responses. To our knowledge, this is the first report of a non-pharmacological effect of perceived cocaine availability on self-reported *baseline *craving prior to receiving cocaine and on subjective and hemodynamic variables following cocaine administration.

The elevated high ratings in the 'unblinded' group, although did not survive stringent statistical adjustment, are suggestive of an expectancy component in cocaine's rewarding effects and are consistent with the greater "high" reports in the context of 100% vs. 50% methylphenidate expectancy state [37]. Although there were important methodological similarities between Volkow et al. [37] and our study (i.e., enrollment of cocaine-dependent subjects, use of subjective self-ratings and acute administration of a psychostimulant), there were also substantial differences in specific definitions of self-ratings, the priming agent, level of uncertainty about the receipt of the drug, intersubject design, and the measures of hemodynamic responses. Thus, these independent results strongly support the validity of the relationship between expectancy context and cocaine effects reflected in self-reports and in physiological changes.

It is notable that notwithstanding similar (to the 'blinded' group) craving scores on a multidimensional assessment several hours before the infusions, 'unblinded' subjects reported higher craving levels on a more proximal evaluation, immediately prior to the infusions. These data are consistent with a substantial body of literature [38-40] documenting failure to detect a relationship between measures of craving obtained in hospital and laboratory environments (where expectancy of obtaining drugs is low) and subsequent relapse to seeking and consuming cocaine. Thus, this study suggests that even within a laboratory environment, craving can be exacerbated by the imminent availability of the drug. Furthermore, our data may be indicative that the effect of expectancy is time sensitive such that proximal rather than distant prospects of getting the drug have greater effect on craving. Therefore, in order to draw valid conclusions about clinical outcomes, laboratory-based research may need to take into account motivational and cognitive contexts of craving e.g., perceived availability of the drug.

Probability of 100% for receipt of cocaine obviously presents a stronger drug-related cue, as compared to 33%, for the sensitized dopaminergic system, thus evoking heightened anticipatory craving. This difference in craving response levels off because the next cue i.e., the priming dose of cocaine itself is not different between the groups. Given the intuitively obvious liking of the desired objects [41], subjects with elevated craving for cocaine, as those in the 'unblinded' condition, presented heightened hedonic rush responses. Alternatively, these may be reflective of the hemodynamic effects (e.g., perception of the elevated heart rate).

Subjective and hemodynamic cocaine effects may indeed be connected via conditioned mechanisms. Thus, heart rate elevations repeatedly paired with cocaine use may become a conditioned stimulus and elicit cocaine-like psychological responses [42,43], which in turn may produce increases in cardiovascular activity [44]. Such a self-sustaining feed-forward loop whereby minor stimuli can trigger escalating reactions may be implicated in both, the chronically deteriorating course of cocaine dependence [45] and in high rates of cardio- and cerebrovascular morbidity associated with this illness [46]. Notably, that causality does not run in the opposite direction as pharmacologically induced decrements in hemodynamic indices do not seem to affect subjective effects of cocaine [47,48].

Homeostatic regulation of hemodynamic function can be dissected into distinct, but interacting catecholaminergic systems including dopamine, norepinephrine and epinephrine. Dopamine has greater β than α affinity, which explains its predominant heart rate effects. On the other hand, a rather selective α agonist, norepinephrine, primarily increases blood pressure, accompanied by only minimal heart rate changes. Epinephrine binds both α and β adrenoceptors, thus evenly affecting blood pressure and heart rate [49]. Hence, different slopes of heart rate increases in the 'unblinded' subjects may reflect an expectancy-induced surge in dopamine [50,51], which is the key neurochemical implicated in expectancy [13,19,20,52] and in sensitization [53] effects.

An alternative/complimentary explanation may be consistent with an idea that a psychological state, such as cocaine expectancy, could alter the homeostatic baroreflex, which is responsible for slowing the heart rate during increases in blood pressure by relaying neural stimuli generated by the arterial receptors' distortion to the CNS, nucleus of the solitary tract [49,54]. Future research can evaluate signs of diminished baroreceptor function such as orthostatic hypotension by performing supine and upright blood pressure measurements along with Valsalva maneuver and plasma catecholamine assays.

This study's strengths include fully randomized and counterbalanced cocaine administration and employment of both subjective and objective measures of cocaine response. There are, however, some caveats that have to be considered in interpreting the present data. First, a limitation of the present study is that group differences in age required us to perform statistical adjustments for this potentially confounding variable. Second, the lack of true randomization into the 'unblinded' and 'blinded' groups, along with the modest size of the latter, render our results preliminary pending replication in more rigorously designed trials.

Third, although the crossover design in the 'blinded' group offers certain advantages [55,56], a few effects could have carried over from the hydrocortisone session. This is unlikely, though, given only transient and subtle subjective effects of hydrocortisone [34] and similar baseline values of the reported variables (excluding craving). Nonetheless, to fully account for such confounding, a second placebo arm could be an important consideration for future research. Fourth, mostly men participated in the experiment and results may not be easily extrapolated to women, as gender differences in craving have been previously demonstrated [57]. Finally, to accommodate for motivational and attentional deficits commonly encountered in cocaine dependent populations [8,58-61], this study focused on short-term acute subjective responses. As some of the self-ratings did not return to baseline at the conclusion of the 20-minute data collection period, longer study duration may have yielded different results.

## 5. Conclusion

In conclusion, rather than being a unitary state characterized by one set of motivations [62], cocaine craving is modulated by its own availability, which also affects other emotions evoked by the drug and cocaine-induced hemodynamic changes.

The inability to correlate craving and subsequent drug taking behavior, as has been shown for laboratory settings, potentially generalizes to hospital and clinical treatment facilities [34,63]. A better model could be evaluations performed at a site with a greater likelihood of obtaining the drug. Thus, current results suggest that more ecologically valid assessments of craving, such as phone interviews in a patient's home, might be important for understanding the patterns of craving that lead to drug use and subsequently for intervention and prevention techniques as well as for proper disease staging and treatment matching procedures employed in public health policy research and determination [64,65].

## Competing interests

The author(s) declare that they have no competing interests.

## Authors' contributions

RY carried out the statistical analysis and drafted the manuscript. KK coordinated the study and carried out the procedures. DR contributed cardiovascular expertise and helped drafting the manuscript. SL helped with the conceptualization and interpretation of the findings. IE designed the study and supervised the co-authors in their writing of the manuscript.  All authors read and approved the final manuscript.
